# Public understanding of palliative care and preferences for place of end-of-life care and death: A national population-based latent class analysis

**DOI:** 10.1177/26323524261474030

**Published:** 2026-07-29

**Authors:** Cecilia Larsdotter, Stina Nyblom, Henrik Imberg, Richard Sawatzky, Joakim Öhlén

**Affiliations:** 1 Department of Nursing Sciences, 25548 Sophiahemmet University, Stockholm, Sweden; 2 Sahlgrenska Academy, Institute of Health and Care Sciences, 70712 University of Gothenburg, Gothenburg, Sweden; 3 Centre for Person-Centred Care (GPCC), Sahlgrenska Academy, 70712 University of Gothenburg, Gothenburg, Sweden; 4 Palliative Centre, 56749 Sahlgrenska University Hospital, Gothenburg, Sweden; 5 Sahlgrenska Academy, Insitute of Medicine, 70712 University of Gothenburg, Sweden; 6 Department of Molecular and Clinical Medicine, Sahlgrenska Academy, Institute of Medicine, 70712 University of Gothenburg, Gothenburg, Sweden; 7 Statistiska Konsultgruppen Sweden, Gothenburg, Sweden; 8 School of Nursing, 4402 Trinity Western University, Langley, BC, Canada; 9 Centre for Advancing Health Outcomes, Providence Health Care Research Institute, Vancouver, BC, Canada; 10 Faculty of Nursing, University of Alberta, Edmonton, AB, Canada

**Keywords:** end-of-life, latent class analysis, public health, literacy, place of death, palliative care, patient preferences, health policy

## Abstract

**Background:**

Dying in the preferred place is associated with improved wellbeing. Preferences may be shaped by personal characteristics, health, prior experiences, and understanding of palliative care.

**Objectives:**

To investigate preferences for place of end-of-life care and death in the Swedish adult population and specifically, to identify subgroups characterised by different understanding of palliative care and examine how preferences vary across these subgroups.

**Design:**

This study was based on a cross-sectional population-level survey.

**Methods:**

A simple random sample of 3,750 16–90-year-old individuals, selected from the Swedish Population Register. Latent class analysis identified distinct subgroups based on participants’ understanding of palliative care. Predictors of subgroup membership were examined using multinomial logistic regression.

**Results:**

A total of 1,752 individuals responded (48%). Of them, 59.6% preferred end-of-life care at home, and 54.2% preferred home death. Latent Class Analysis identified five distinct subgroups: comprehensive understanding, some understanding, limited understanding, misunderstanding, and no opinion. Comprehensive understanding, such as believing that palliative care supports families and alleviates suffering, was associated with preferences for home or hospice care. Misunderstanding, such as believing that palliative care hastens death, was associated with preferences for hospital or nursing home. The comprehensive understanding group included more women (57.6%), older (mean [SD] age: 57 [18] years), and university-educated individuals (48.2%).

**Conclusion:**

Although the most preferred place for both care and death were home, preferences varied across subgroups defined by differing levels of understanding of palliative care and sociodemographic characteristics.

## Plain language summary

Why was the study done? Where people spend their final days of life, whether at home, in hospital, or elsewhere can affect their wellbeing at the end of life. Many things influence people’s preferences for place of end-of-life care and death, including their understanding of palliative care. The researchers wanted to understand where adult people in Sweden prefer to receive care and die. They also wanted to identify groups with different levels of understanding of palliative care and see how their preferences vary. What did the researchers do? They sent a survey to a random representative sample of adults across Sweden. The survey asked about preferences for where they would like their end-of-life care and death to take place, and how much they understood about palliative care. Using a statistical method, the researchers grouped people based on their understanding of palliative care. They then examined which personal characteristics were distinct for each group. What did the researchers find? Out of 3,750 people contacted, 1,752 responded (48%). Most people preferred care (59.6%) and death (54.2%) at home. Five subgroups were identified based on how people understood palliative care: (1) comprehensive understanding; (2) some understanding; (3) limited understanding; (4) misunderstanding; and (5) no opinion. People with comprehensive understanding were more likely to be women, slightly older, and with university education. A good understanding (e.g., recognising that palliative care reduces suffering and supports families) was linked to preferences for home or hospice care. Misunderstandings (e.g., thinking palliative care hastens death) were linked to preferring hospital or nursing home care. What do the findings mean? Although home was the most preferred place for care and death, preferences differed based on their understanding of palliative care and their personal characteristics.

## Background

In high-income countries, palliative care discourse increasingly emphasises aligning care with individuals’ preferences regarding where to receive care and where to die.^1^ Dying in the preferred place is associated with improved wellbeing at the end of life.^2^ People’s preferences may be shaped by personal characteristics, health status or previous healthcare experiences. For example, it has been reported that being a woman, well-educated, or a widow/widower or partner of a deceased person are associated with a preference for inpatient palliative care, whereas older adults or those with more long-term chronic diseases lean towards other forms of care, including nursing homes, over home care.^3–8^ Family dynamics,^3,9^ structural vulnerability^10^ or cultural perspectives^3^ may also shape people’s preferences.

Understanding of palliative care (i.e., awareness of its core purpose among stakeholders, health service organisations and the public) has emerged as a key factor in improving how care is understood, accessed, and delivered across health systems.^11,12^ Understanding palliative care empowers people to make informed decisions and advocate for appropriate services. It also provides a means of exerting pressure to ensure equal opportunities for palliative care.^13^

From a public health palliative care perspective, place of death is considered an indicator of the infrastructure and organisation of palliative care in a country.^14^ The degree to which palliative care is integrated into health systems significantly impacts where people die, as do the infrastructure and organization of palliative care.^13^ Countries with robust primary care and community-based services are often better equipped to support home deaths. Conversely, hospital-centric systems may lead to higher rates of hospital deaths. Countries with well-developed, integrated palliative care services across various settings offer more options for end-of-life care. The availability of comprehensive home-based palliative care services can make home deaths more feasible, and early integration of palliative care has been associated with increased likelihood of dying at home.^3,15,16^

In Sweden, which is the setting of this study, palliative care is highly integrated, with national policy documents implemented since 2013.^17,18^ These documents propose a person-centred approach to palliative care that aims to address patients’ needs throughout their illness trajectory, meaning it should be available in all care places where people die, including at home. It emphasises the patients’ right to participate in decision-making about care but lacks a clear statement about achieving the preferred place for end-of-life care and death.

In many high-income countries, home is the preferred place of death for most people.^9,19,20^ In Sweden, similar patterns have been reported in studies of bereaved family members as well as in population-based samples of adults.^8,21^ In low-income countries and in settings with limited health insurance coverage, home-based care may be preferred due to financial constraints.^9^

Identifying preferences for end-of-life care and death and the factors that shape these preferences is essential for understanding population needs and informing resource allocation and service planning. Furthermore, recognising distinct subgroups based on these preferences provides a foundation for developing public health-oriented interventions and implementation strategies that align with policy goals, specifically, ensuring equitable access to person-centred care and supporting participation in decisions about how and where to receive end-of-life care.^22^

## Aim and research questions

This study aimed to investigate preferences for place of end-of-life care and place of death in the Swedish adult population, to identify subgroups characterised by differing levels of understanding regarding palliative care, and to examine how preferences vary across these subgroups. Specifically, the study addressed the following questions:

- What subgroups can be identified based on understanding of palliative care?
- What sociodemographic factors are associated with these subgroups?

## Design and methods

This was a population-based, cross-sectional study using a representative sample of the adult population in Sweden.

### Sample

Data were obtained from the Swedish nationwide Society, Opinion, Media (SOM) survey (version R6), conducted in 2023. The survey, which is administered annually by the SOM institute at the University of Gothenburg,^23,24^ was selected for its well-established methodology and high level of national representativeness.^23^ A simple random sample of 3,750, 16–90-year-old individuals was selected from the Swedish Tax Agency’s population register (N = 8,542,047 as of 15 August 2023), which includes all Swedish and foreign citizens with a registered primary address in Sweden. The population register is continuously updated, and coverage errors (e.g., due to death, immigration, or emigration) are minimal. The sample was subsequently verified for age and gender distribution by the SOM institute against official statistics from Statistics Sweden (SCB), confirming representativeness without the need for adjustment or re-sampling. Sample size was determined based on an expected response rate of approximately 50%, ensuring sufficient representation across demographic, socioeconomic, and political groups. The current sample comprised 1,752 respondents from the 3,750 individuals invited to participate.

### Data and variables

The full SOM survey comprised 184 items on matters of societal relevance. Three items related to preferences and understanding of palliative care were added specifically for this study. These items were based on the international PRISMA-survey^25^ and had previously been translated and validated for use in Swedish^8^: *Imagine if you had a serious illness and less than a year to live. If you got the care and support you needed, how much do you agree that you would want to be 1) cared for and 2) die in the following places?* Response options for both questions were: at home; at a friend’s home; in a hospice or an inpatient palliative care unit; in a nursing home; in a hospital but not an inpatient palliative care unit; and somewhere else. Participants rated their agreement with each potential place on a four-point Likert scale (“strongly disagree”, “somewhat disagree”, “somewhat agree”, “strongly agree”), with an additional “no opinion” option. The preferred place of end-of-life care and death was defined as the option(s) with the highest agreement score; when multiple options shared the highest rating, equal weights were assigned. Participants were also asked whether they had prior experience with palliative care as patients, healthcare professionals, family members, or volunteers.

In addition, the survey included sociodemographic variables from the standard SOM questionnaire, covering individual characteristics (age, gender, country of birth, primary language, self-rated health, and life satisfaction); geographic characteristics (type of residential area); and socioeconomic characteristics (education level, employment status, marital status, and living arrangements). Gender was self-reported with three response options; women, men, and other. Self-rated health was assessed on a scale ranging from 0 (very poor) to 10 (very good), and life satisfaction was measured on a four-point scale ranging from 1 (very satisfied) to 4 (very dissatisfied).

To assess how understanding of palliative care was associated with preferences for end-of-life care and place of death, six statements were included in the questionnaire. These were based on established palliative care principles and misconceptions, such as the belief that symptom relief may hasten death:^21,26^ *Palliative care aims to reduce the patient’s suffering; Palliative care hastens death; Palliative care is provided in all types of healthcare and care facilities; Pain is inevitable in the dying process; Morphine at the end of life alleviates pain without hastening death; and Palliative care includes support for family members*. Participants were asked to indicate their level of agreement with the statements on a four-point Likert scale with the options “strongly disagree”, “somewhat disagree”, “somewhat agree”, and “strongly agree”, along with an additional “no opinion” option. For presentation purposes, the categories “strongly disagree” and “somewhat disagree” were combined into a single “disagree” category, and “somewhat agree” and “strongly agree” were combined into a single “agree” category. The original four responses were retained for all statistical analyses. Items are available in Supplementary file 1.

The survey was distributed by post, with the option to respond and return the completed questionnaire online or by post. Three postal and text-message reminders were sent to non-respondents, each including a paper questionnaire and login details for the online response option. Consent was considered obtained when respondents completed and submitted the questionnaire.

The study followed the 2024 revised Helsinki declaration. Approval was obtained from the Swedish Ethical Review Authority (File no.2021-06242-01).

### Statistical analyses

Descriptive statistics were summarised using means and standard deviations (SDs) for continuous variables, medians, and interquartile ranges (IQRs) for ordinal variables, and counts with percentages for other categorical variables. To assess representativeness, characteristics of the study sample were compared with those of the total population (16–90 years of age) with respect to age, gender, and citizenship, based on data from the Swedish Tax Agency’s population register.^24^

To identify subgroups based on individuals’ level of understanding regarding palliative care, latent class analysis (LCA) was performed based on participants’ responses to the six statements.^27^ Responses were treated as categorical variables, with the “no opinion” option retained to reflect uncertainty or limited knowledge. Missing data were handled using random forest imputation. The optimal number of classes was determined based on the lowest Bayesian Information Criterion (BIC). Model fit was assessed using entropy, which quantifies classification certainty. In accordance with general guidelines, values close to or above 0.80 were considered indicative of acceptable class separation.

Associations between individual, geographic, and socioeconomic characteristics and subgroup membership were evaluated using multivariable multinomial logistic regression, with subgroup membership as the outcome. Estimation was conducted using a bias-adjusted three-step procedure, weighted by posterior subgroup probabilities to account for classification uncertainty.^28^ In this approach, the measurement model was first estimated, followed by subgroup assignment and then regression analysis, with measurement parameters held fixed to preserve the latent structure.

Statistical analyses were conducted using R software, version 4.2.3 (R Foundation for Statistical Computing, Vienna, Austria). LCA was performed using the poLCA package (version 1.6.0.1).

## Results

### Sample characteristics

Of the 3,750 invited to participate, 1,752 completed the survey, yielding a response rate of 47%. The mean (SD) age of participants was 55 (20) years. Regarding gender, 51.4% identified as women, 45.3% as men, 1.4% as another gender, and 1.9% did not respond. Most participants (82%) had completed at least secondary education, and 89% had Swedish citizenship only. The median (IQR) self-rated health score was 8 (6–9) on a 10-point scale, with higher scores indicating better health, and the life satisfaction score was 2 (1–2) on a 4-point scale, with lower scores indicating greater satisfaction. Overall, 42% of participants reported previous experience with palliative care. Further details are provided in Table1.

**Table 1.** Sociodemographic and health-related characteristics of the study population (n=1,752).

​	Mean (SD)/Median (IQR) or n (%)	n missing
Gender		33
Women	900 (51.4%)	​
Men	794 (45.3%)	​
Other	25 (1.4%)	​
Age (years), mean (SD)	55 (20)	0
Citizenship		
Swedish	1,567 (89.4%)	​
Swedish and another country	80 (4.6%)	​
Other country	60 (3.4%)	​
Swedish as primary language	1,543 (88.1%)	106
Education level		57
Primary (9 years)	250 (14.3%)	​
Secondary (12 years)	721 (41.2%)	​
University (>12 years)	724 (41.3%)	​
Relationship status and living condition		
In a relationship	1,263 (72.1%)	56
Living alone	342 (19.5%)	57
Residential area		74
Small city, or rural area	187 (10.7%)	​
City	910 (51.9%)	​
Big city	581 (33.2%)	​
Work status		60
Employed	875 (49.9%)	​
Unemployed	35 (2.0%)	​
Retired	614 (35.0%)	​
Long-term sick leave	38 (2.2%)	​
Student	148 (8.4%)	​
Other	47 (2.7%)	​
Overall health status*, median (IQR)	8 (6–9)	68
Overall life satisfaction^†^, median (IQR)	2 (1–2)	58
Personal experience with palliative care	712 (42.1%)	61

*Notes.* Descriptive data are presented as means with standard deviation (SDs) for continuous variables, medians with interquartile ranges (IQRs) for ordinal variables, and counts and percentages for other categorical variables.

Percentages are calculated using the total study sample (n = 1,752) as the denominator, including participants with missing data; counts of missing observations are reported separately.

*Overall health status was rated on a scale from 0 (very poor) to 10 (very good).

^†^Overall life satisfaction was rated on a scale from 1 (very satisfied) to 4 (very dissatisfied).

Abbreviations: IQR, interquartile range; SD, standard deviation.

Compared with the total adult population, the study sample included a similar proportion of women (51% in the sample vs 50% overall), fewer individuals aged 16–29 (14% vs 20%) and 30–49 years (26% vs 33%), and more aged 65–90 years (36% vs 25%).

### Palliative care understanding and preferences for place of care and death

Most participants (79%) agreed that palliative care aims to reduce suffering, and 57% that it includes support for family members. Only 26% agreed that it is provided in all healthcare settings, and 23% believed that pain is inevitable in the dying process. More than half (59%) agreed that morphine alleviates pain without hastening death, while 16% believed that palliative care hastens death. The proportion of participants reporting lack of awareness or not taking a stand (“no opinion”) ranged from 17% to 55%, with the highest proportions for the statements that palliative care is provided in all healthcare settings (55%), that it includes support for family members (35%), that morphine alleviates pain without hastening death (30%), and that palliative care hastens death (30%) (Table 2).

**Table 2.** Understanding of palliative care among participants (n=1,752).

​	Strongly disagree	Somewhat disagree	Somewhat agree	Strongly agree	No opinion	n missing
Palliative care aims to reduce the patient’s suffering	28 (1.7%)	26 (1.6%)	269 (16.2%)	1,048 (63.1%)	289 (17.4%)	92
Palliative care hastens death	541 (33.0%)	339 (20.7%)	201 (12.3%)	65 (4.0%)	492 (30.0%)	114
Palliative care is provided in all types of healthcare and care facilities	156 (9.6%)	168 (10.3%)	263 (16.1%)	155 (9.5%)	887 (54.5%)	123
Pain is inevitable in the dying process	494 (30.4%)	303 (18.6%)	285 (17.5%)	93 (5.7%)	452 (27.8%)	125
Morphine at the end of life alleviates pain without hastening death	89 (5.4%)	87 (5.3%)	415 (25.2%)	560 (34.0%)	495 (30.1%)	106
Palliative care includes support for family members	61 (3.7%)	68 (4.2%)	337 (20.6%)	595 (36.4%)	575 (35.1%)	116

*Note.* Descriptive data are presented as counts and row percentages among respondents, excluding missing values. The number of missing responses is shown in the final column.

Most participants expressed the highest preferences for receiving end-of-life care (59.6%) and for dying (54.2%) at home. Most participants also strongly agreed that home was the preferred place for care (63.1%) and place of death (54.9%). The second most preferred setting was a hospice or an inpatient palliative care unit, chosen by 11.5% for care and 14.7% for death. Only 6.2% preferred to receive care, and 7.7% to die, in a hospital. Similarly, 7.9% preferred care and 7.7% death in a nursing home. Another location (“somewhere else”) was selected by 4.1% as the preferred place for end-of-life care and by 5.6% as the preferred place of death (Table 3).

**Table 3.** Distribution of preferences for end-of-life-care and place of death in the study population (n=1,752).

​	Strongly disagree	Somewhat disagree	Somewhat agree	Strongly agree	No opinion	Missing, n
**Preferred place of care at the end of life**						
At home	61 (3.7%)	33 (2.0%)	379 (22.8%)	1,048 (63.1%)	141 (8.5%)	90
At a friend’s home	534 (33.0%)	230 (14.2%)	360 (22.3%)	272 (16.8%)	220 (13.6%)	136
In hospice or an inpatient palliative care unit	327 (20.2%)	242 (15.0%)	524 (32.4%)	237 (14.7%)	285 (17.6%)	137
In hospital	430 (26.6%)	319 (19.8%)	397 (24.6%)	125 (7.7%)	343 (21.3%)	138
In a nursing home	468 (28.7%)	316 (19.4%)	385 (23.6%)	176 (10.8%)	287 (17.6%)	120
Somewhere else	375 (23.8%)	76 (4.8%)	122 (7.7%)	84 (5.3%)	921 (58.4%)	174
**Preferred place of death**						
At home	112 (6.8%)	54 (3.3%)	318 (19.3%)	904 (54.9%)	258 (15.7%)	106
At a friend’s home	596 (37.2%)	162 (10.1%)	264 (16.5%)	237 (14.8%)	341 (21.3%)	152
In hospice or an inpatient palliative care unit	376 (23.4%)	171 (10.7%)	427 (26.6%)	267 (16.6%)	363 (22.6%)	148
In hospital	475 (29.7%)	230 (14.4%)	349 (21.8%)	144 (9.0%)	403 (25.2%)	151
In a nursing home	544 (33.7%)	230 (14.3%)	314 (19.5%)	150 (9.3%)	376 (23.3%)	138
Somewhere else	412 (26.4%)	60 (3.8%)	120 (7.7%)	112 (7.2%)	859 (55.0%)	189

*Notes.* Descriptive data are presented as counts and row percentages, excluding missing values.

Questions on preferences were phrased as: *“Imagine if you had a serious illness and less than a year to live. If you got the care and support you needed, how much do you agree that you would want to receive care in the following places?”* and *“Imagine if you had a serious illness and less than a year to live. If you got the care and support you needed, how much would you agree that you would want to die in the following places?”*

* Preferred place of care and death was defined as the location(s) with the highest level of agreement on the Likert scale, assigning equal weights when ties occurred. Abbreviations: NA, not applicable.

### Subgroups based on palliative care understanding

**Figure 1.**
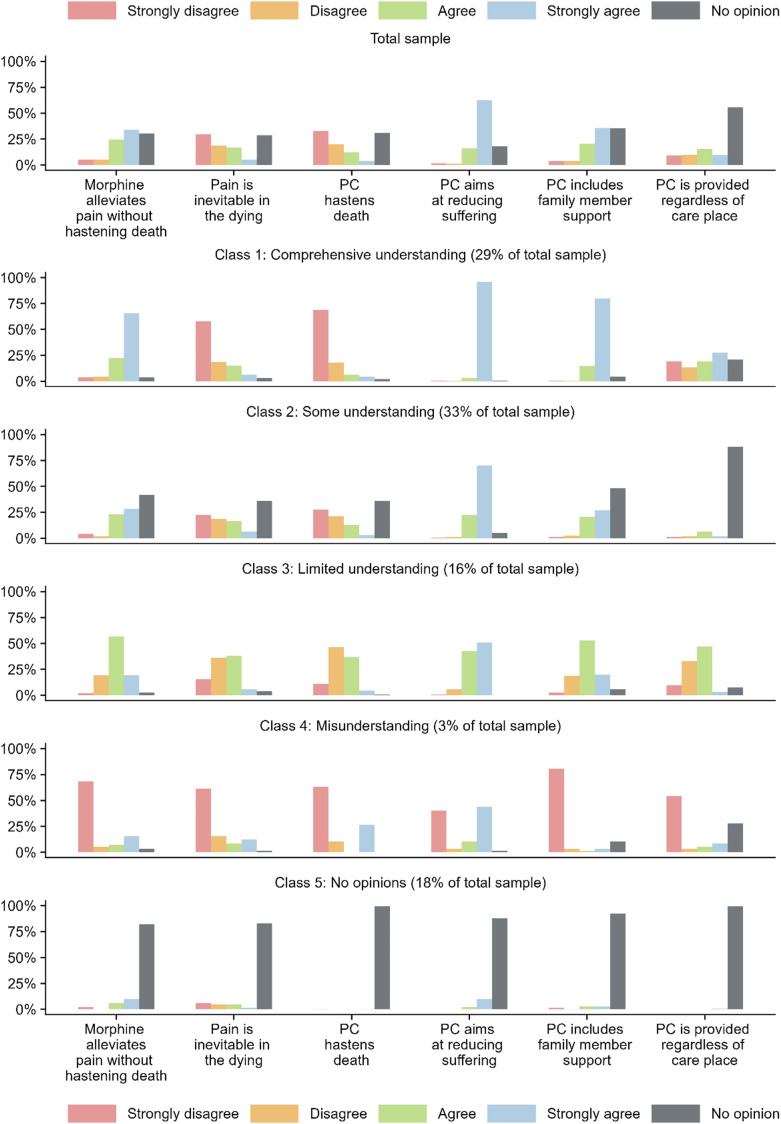
Understanding of palliative care in the total sample and across subgroups. *Note.* Responses to six statements about palliative care are displayed as proportions across response categories: strongly disagree, somewhat disagree, somewhat agree, strongly agree, and no opinion. Each panel corresponds to one latent class (i.e., subgroup), labelled according to the level of understanding and its proportion of the total sample. PC = palliative care.

To identify distinct subgroups (i.e., latent classes) of participants based on their understanding of palliative care, a five-group model was selected as the best-fitting solution, based on the lowest Bayesian Information Criterion (BIC) (Supplementary file 2, Table S1 and Figure S1). The following five subgroups were identified: 1) *Comprehensive understanding* (n=507), consisting of participants with the most informed views; 2) *Some understanding* (n=584), including participants that were generally informed but showed less specific knowledge, reflected in a higher proportion of “no opinion” responses; 3) *Limited understanding* (n=283), comprising participants with overall uncertain and inconsistent responses across statements; 4) *Misunderstanding* (n=57), including participants who held predominantly incorrect views, such as believing that palliative care hastens death; and 5) *No opinion* (n=321), characterised by limited engagement or awareness, with many selecting “no opinion” across multiple items (Figure 1 and Supplementary file 2, Table S2). The model achieved an entropy of 0.77, reflecting moderate class separation.

### Correlates of subgroup memberships

**Figure 2.**
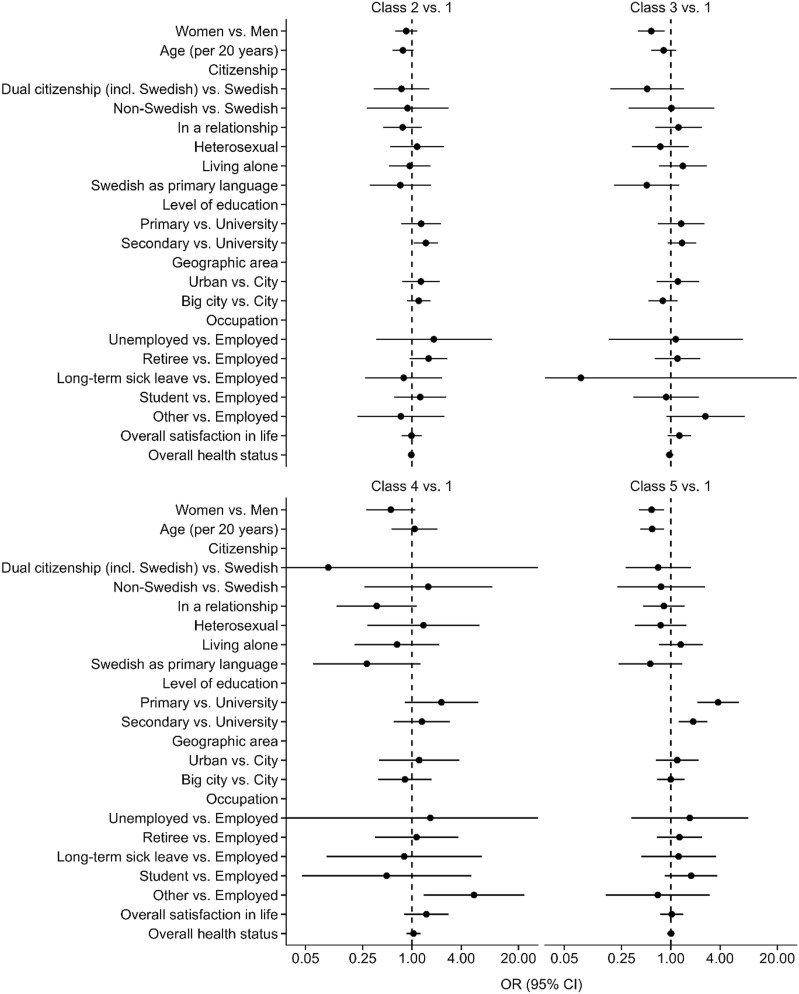
Associations between sociodemographic characteristics and latent class membership, with Class 1 (comprehensive understanding) as the reference. Points and error bars represent odds ratios (ORs) and 95% confidence intervals (CIs) for Classes 2–5 compared to Class 1. ORs >1 indicate a higher likelihood and ORs <1 a lower likelihood of membership relative to Class 1. Class 2 = Some understanding; Class 3 = Limited understanding; Class 4 = Misunderstanding; Class 5 = No opinion. For life satisfaction, higher values indicate lower satisfaction (1 = very satisfied, 4 = very dissatisfied). For self-rated health, higher values indicate better perceived health (0 = very poor, 10 = very good). Gender was included as a three-category variable (women, men, another gender). Due to small numbers in the “other” category, estimates are presented for women versus men only, and the “other” category is not shown separately.

Sociodemographic and health-related characteristics related to subgroups are shown in Figure 2 and Supplementary file 2, Table S3 and S4. Members of the *comprehensive understanding* subgroup (Class 1) included more women (306/507; 60.4%), were older on average (mean [SD] age: 57 [17] years), and had a greater proportion with a university education (257/507; 50.7%) compared to the overall sample (Supplementary file 2, Table S3).

Compared with the *comprehensive understanding* group (Class 1; reference group), participants with *some understanding* (Class 2) had lower educational attainment (OR [95% CI] 1.48 [1.05–2.07] for secondary vs university education. Those with *limited understanding* (Class 3) were more often men (OR 0.58 [0.40–0.84] for women vs men). The *misunderstanding* group (Class 4) included fewer women (OR 0.55 [0.28–1.10] for women vs men), and more participants with lower education (OR 2.29 [0.82–6.43] for primary vs university education), although the sample size in this class was small (n=57) and differences by gender and education were not statistically significant. Those in the *no-opinion* group (Class 5) were generally older (OR 0.59 [0.42–0.83] per 20-year increase), more often men (OR 0.58 [0.41–0.83] for women vs men), and more likely to have lower educational levels (OR 3.78 [2.10–6.79] and OR 1.88 [1.25–2.81]) for primary vs university and secondary vs university education, respectively), compared with the *comprehensive understanding* group (Figure 2 and Supplementary file 2, Table S4).

### Preferences for place of death by subgroup membership

**Figure 3.**
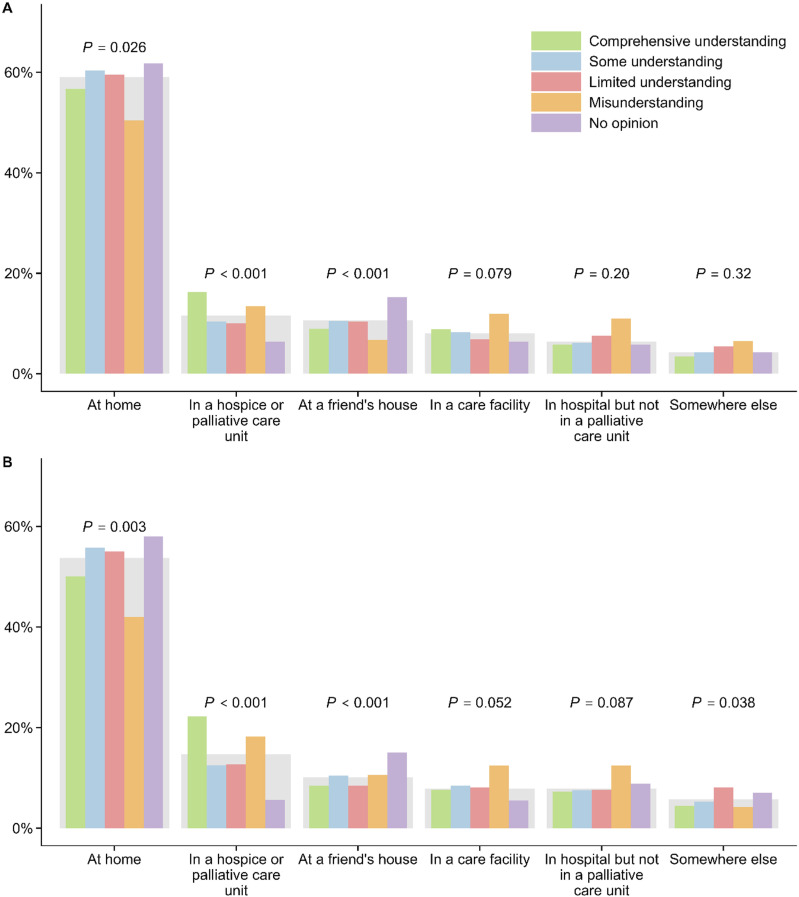
Preferred place of (a) end-of-life care and (b) place of death by subgroup membership *Notes:* Grey bars in the background indicate preferences in the overall sample. When participants selected more than one preferred place (i.e., assigned the same highest score to multiple options on the Likert scale), responses were weighted equally across the selected categories. *P*-values represent differences in preference rates across subgroups, assessed using regression models for fractional outcomes (two-sided test).

Home was the most preferred place for end-of-life care and death across all subgroups. However, participants in the *comprehensive understanding* group were more likely to prefer care and death in a hospice or palliative care unit. Participants in the *no-opinion* group expressed a higher preference for being cared for and dying at a friend’s home. By contrast, those in the *misunderstanding* group were less likely to prefer home and more likely to prefer a nursing home or a hospital setting outside a palliative care unit (Figure 3, Supplementary file 2, Table S5).

## Discussion

This study demonstrates that although most participants preferred to receive end-of-life care and die at home, preferences were heterogeneous across the population. We identified five subgroups based on the participants’ understanding of palliative care. These were associated with differing preferences for place of care and death, with some or comprehensive understanding linked to a higher likelihood of preferring care and death at home or in hospice, and less understanding associated with preferences for hospital or nursing home settings. Subgroup membership was also significantly associated with sociodemographic characteristics, including age, gender, and educational attainment. These findings are consistent with reports from several countries,^3–8^ although variations do also exist. For example, in a public Chinese sample, fewer than one-fifth stated a preference for home death,^29^ and similarly, in a New Zealand sample of older adults, dying at home was a low priority among other end-of-life priorities.^30^ In a recent review, people’s preferences for a home death were found to often involve motives such as dignity preservation; comfort and safety; maintaining normality; being close to social networks; and prior negative experiences of hospital care.^9^ One of our previous studies based on a smaller sample with bereaved family members indicated that preferences were influenced by previous experience of palliative care,^8^ but this was not a significant factor in the current study. As previously mentioned, preferences for home care and dying at home may also vary across countries according to the strength of supportive welfare systems.^9^ Sweden’s tax-funded healthcare system is characterised by decentralisation and a non-directive policy approach.^31^ The Ministry of Health sets national policy, while regional councils and municipalities independently finance and provide services.^17^ Palliative care is delivered across primary care, home care, hospitals, and nursing homes. Costs of care (including end-of-life care) are covered through taxation, regardless of the care setting. This reduces the likelihood that preferences for home-based care or home death are driven by financial constraints.

A long-running reform in Sweden seeks to promote integrated, person-centred care closer to people’s homes,^32^ which may, in principle, benefit peoples’ preferences for home-based care and dying at home. Since the implementation of national palliative care policy in 2013, there has been a slight decrease in the proportion of hospital deaths and conversely a small increase in the proportion of home deaths. However, this trend is not consistent across regions^33^ and diagnosis groups^33–36^; and in 2019, of the approximately 85,000 annual adult deaths, still only one in five occurred at home, while two in five occurred in hospitals — with another two in five occurring in nursing homes.^33^ It is likely that the disparity in public understanding exacerbates inequities in access to hospice- and home-based palliative care.

The results of our study suggest the need for a more progressive approach to articulating preferences regarding place of end-of-life care and death. This requires revision of national policy for conceptual clarity, as well as the systematic implementation of strategies and geographically adjusted organisation of specialised and non-specialised palliative care services. The discrepancy between preferred and actual place of death underscores the importance of person-centred care. Such care ensures that individuals are well informed, supported, and enabled to make meaningful choices about the final phase of life, including how and where end-of-life care is provided and where death occurs.^37^ Consistent with previous research,^3–8^ our study found that people with lower educational attainment —as well as those with limited understanding, misunderstanding, or no opinion about palliative care— are more likely to prefer hospital-based care and death. This is of particular importance since limited understanding or misunderstanding about palliative care among both patients and healthcare professionals are well-established barriers to its early integration in these settings.^26^

Our findings highlight the need for comprehensive, public health–oriented national palliative care strategies. Such strategies should involve requirements regarding the inclusion of palliative care (including public health approaches) in all higher education curricula for health- and social professions and the implementation of systematic and recurring clinical palliative care training in all relevant care places, based on standardised national educational materials. Public education initiatives, including targeted campaigns for school children, youth and adults, including retirees, should also be initiated, to improve societal understanding and engagement with palliative care.^38–40^

A decentralised health system like the Swedish risks making end-of-life support fragmented and unequal across settings and providers^41^ A public health palliative care compassionate communities’ model may counteract or at least complement this.^40^ Its core contributions are to promote societal awareness and acceptance of death and dying by building public understanding of palliative care and its role, and growing sustainable, network-based caring capacity through community development, to improve equity of access to end-of-life care.^31^ Finally, public health palliative care strategies should include clear specifications and requirements for continuous, measurable evaluation, aligned with strengthened national palliative care policies.

### Study limitations

The study sample included an overrepresentation of older and Swedish-born participants, which may limit the generalisability of the findings to younger age groups and people with immigrant backgrounds. In addition, the response rate was relatively low (∼50%), and the possibility of non-response bias cannot be ruled out.

The statements used to assess understanding of palliative care have not previously been validated or applied in their exact format. However, the statements are consistent with prior research about knowledge gaps and misunderstandings about palliative care that are commonly expressed among both the public and patients. The subgroups were identified using a data-driven approach. While they reflect meaningful variation in palliative care understanding within this sample, different or additional subgroups could emerge in other populations or contexts. The cross-sectional design also restricts the ability to draw conclusions about causality; associations between palliative care understanding and care preferences should be interpreted accordingly. A larger sample may also result in other subgroups being identified. Nonetheless, although an a priori power analysis was not conducted, the sample size exceeds conventional recommendations for latent class analyses requiring a minimum of 500 participants.^27^

### Conclusion and implications for policy and practice

This study revealed marked differences in how palliative care is understood among the public. Although home was the most preferred place for both care and death, preferences differed across groups depending on the level of palliative care understanding and demographic characteristics such as age, gender, and education. The results can inform policy and service planning to ensure that end-of-life care is aligned with the diverse values and expectations of the population.

## Supplemental material

Supplemental material - Public understanding of palliative care and preferences for place of end-of-life care and death: A national population-based latent class analysis Supplemental material for Public understanding of palliative care and preferences for place of end-of-life care and death: A national population-based latent class analysis by Cecilia Larsdotter, Stina Nyblom, Henrik Imberg, Richard Sawatzky, and Joakim Öhlén in Palliative Care and Social Practice.

Supplemental material - Public understanding of palliative care and preferences for place of end-of-life care and death: A national population-based latent class analysis Supplemental material for Public understanding of palliative care and preferences for place of end-of-life care and death: A national population-based latent class analysis by Cecilia Larsdotter, Stina Nyblom, Henrik Imberg, Richard Sawatzky, and Joakim Öhlén in Palliative Care and Social Practice.

## Data Availability

We acknowledge the use of data and materials made freely available under the Creative Commons Attribution (CC BY) licence, which permits unrestricted use, distribution and reproduction, provided that the original authors are credited. The original dataset used for this study is available from the Swedish National Data service (SND) — a national repository for research data at: https://www.gu.se/en/som-institute/the-som-surveys/data#data-from-the-som-surveys.
