# Supplementary material for: Public understanding of palliative care and preferences for place of end-of-life care and death: A national population-based latent class analysis
Source: Palliat Care Soc Pract. 2026 Jul 29;20:26323524261474030. doi: 10.1177/26323524261474030 (PMC13424512; doi:10.1177/26323524261474030)
Supplement: Supplemental material - Public understanding of palliative care and preferences for place of end-of-life care and death: A national population-based latent class analysis [file sj-pdf-1-pcr-10.1177_26323524261474030.pdf]

Supplementary file 2, questionnaire items used in the study

The full nationwide SOM survey (version R6, 2023) comprises 184 items on matters of societal relevance. Below, the items used in this study are presented in Swedish along with their English translations (made for communication purpose only). Items f147–f150 relate to preferences and understanding of palliative care and were added specifically for this study.

Items f147-f148 were based on the international PRISMA-survey and have previously been translated and validated for use in Swedish. Items f149-f150 were based on established palliative care principles and misconceptions, such as the belief that symptom relief may hasten death. References are provided in the main manuscript.

The remaining items were selected from the SOM questionnaire, covering individual characteristics (age, gender, country of birth, primary language, self-rated health, and life satisfaction); geographic characteristics (type of residential area); and socioeconomic characteristics (education level, employment status, marital status, and living arrangements).

The full Questionnaire (in Swedish) is available at: https://www.gu.se/sites/default/files/2024-09/SOMKodbok_2023.pdf

ACTIVITIES, INTERESTS, VALUES

f109 Hur nöjd är du på det hela taget med det liv du lever?

Mycket nöjd	Ganska nöjd	Inte särskilt nöjd	Inte alls nöjd
☐	☐	☐	☐
**1**	**2**	**3**	**4**

In English: How satisfied are you, overall, with the life you live?

☐ Very satisfied

☐ Fairly satisfied

☐ Not very satisfied

☐ Not at all satisfied

RESEARCH AND PUBLIC HEALTH

f142 Hur bedömer du ditt allmänna hälsotillstånd?

In English: How would you rate your general health?

Very poor

Very

Mycket dåligt

Mycket gott

0	1	2	3	4	5	6	7	8	9	10
☐	☐	☐	☐	☐	☐	☐	☐	☐	☐	☐
**0**	**1**	**2**	**3**	**4**	**5**	**6**	**7**	**8**	**9**	**10**

* **f147 Tänk dig en situation där du har en allvarlig sjukdom med mindre än ett år kvar att leva. Om du får den vård och stöd du behöver, hur väl instämmer du i att du skulle vilja vårdas på följande platser?**

	Instämmer inte alls	Instämmer knappast	Instämmer delvis	Instämmer helt	Ingen uppfattning
**a** Hemma	☐	☐	☐	☐	☐
**b** Hemma hos en närstående/en vän	☐	☐	☐	☐	☐
**c** På hospice eller palliativ vårdavdelning	☐	☐	☐	☐	☐
**d** På sjukhus men inte på palliativ vårdavdelning	☐	☐	☐	☐	☐
**e** På ett vård- och omsorgsboende	☐	☐	☐	☐	☐
**f** Någon annanstans	☐	☐	☐	☐	☐
	**1**	**2**	**3**	**4**	**5**

In English: Imagine if you had a serious illness and less than a year to live. If you got the care and support you needed, how much do you agree that you would want to be cared for in the following places?

	Strongly disagree	Somewhat disagree	Somewhat agree	Completely agree	No opinion
a. At home					
b. At a friend's home					
c. In hospice or an inpatient palliative care unit					
d. In hospital					
e. In a nursing home					
f. Somewhere else					
	1	2	3	4	5

* **f148 Tänk dig samma situation, hur väl instämmer du i följande påståenden? Jag skulle vilja dö...**

	Instämmer inte alls	Instämmer knappast	Instämmer delvis	Instämmer helt	Ingen uppfattning
**a** ... Hemma	☐	☐	☐	☐	☐
**b** ... Hemma hos en närstående/en vän	☐	☐	☐	☐	☐
**c** ... På hospice eller palliativ vårdavdelning	☐	☐	☐	☐	☐
**d** ... På sjukhus men inte på palliativ vårdavdelning	☐	☐	☐	☐	☐
**e** ... På ett vård- och omsorgsboende	☐	☐	☐	☐	☐
**f** ... Någon annanstans	☐	☐	☐	☐	☐
	**1**	**2**	**3**	**4**	**5**

In English: Imagine if you had a serious illness and less than a year to live. If you got the care and support you needed, how much do you agree that you would want to die in the following places?

	Strongly disagree	Somewhat disagree	Somewhat agree	Strongly agree	No opinion
a. At home					
b. At a friend's home					
c. In hospice or an inpatient palliative care unit					
d. In hospital					
e. In a nursing home					
f. Somewhere else					
	1	2	3	4	5

* **f149 I vilken utsträckning instämmer du i följande påståenden?**

	Instämmer inte alls	Instämmer knappast	Instämmer delvis	Instämmer helt	Ingen uppfattning
**a** Palliativ vård syftar till att minska patientens lidande	☐	☐	☐	☐	☐
**b** Palliativ vård påskyndar döden	☐	☐	☐	☐	☐
**c** Palliativ vård bedrivs inom alla typer av vård- och omsorgsenheter	☐	☐	☐	☐	☐
**d** Smärta är oundvikligt i döendet	☐	☐	☐	☐	☐
**e** Morfin i livets slut lindrar smärta utan att påskynda döendet	☐	☐	☐	☐	☐
**f** Palliativ vård inkluderar stöd till närstående	☐	☐	☐	☐	☐
	**1**	**2**	**3**	**4**	**5**

In English: To what extent do you agree with the following statements?

	Strongly disagree	Somewhat disagree	Somewhat agree	Strongly agree	No opinion
a. Palliative care aims to reduce the patient's suffering					
b. Palliative care hastens death					
c. Palliative care is provided in all types of healthcare and care facilities					
d. Pain is inevitable in the					

dying process					
e. Morphine at the end of life alleviates pain without hastening death					
f. Palliative care includes support for family members					
	1	2	3	4	5

*** f150 Har du egen erfarenhet av palliativ vård?**

a ☐ Ja, som patient
b ☐ Ja, som närstående
c ☐ Ja, som personal
d ☐ Ja, som volontär
e ☐ Ja, i annan roll
f ☐ Nej
g ☐ Vet ej

In English: Do you have personal experience of palliative care?

a. ☐ Yes, as patient
b. ☐ Yes, as family member
c. ☐ Yes, as healthcare professional
d. ☐ Yes, as volunteer
e. ☐ Yes, in another role
f. ☐ No
g, ☐ Don´t know

EMPLOYMENT

f155 Vilken av de här grupperna tillhör du för närvarande?

a ☐ Förvärvsarbetande (även sjukskriven, föräldraledig)
b ☐ Har arbete i arbetsmarknadspolitiska åtgärder/ genomgår arbetsmarknadsutbildning
c ☐ Arbetslös
d ☐ Ålderspensionär/avtalspensionär
e ☐ Har sjuk-/aktivitetsersättning
f ☐ Studerande
g ☐ Annat: ....**angrupp**...................

In English: Which of these groups do you currently belong to?

a ☐ Employed (including on sick leave or parental leave)
b ☐ In employment support programs / undergoing labour market training
c ☐ Unemployed
e ☐ Receiving sickness benefit / disability benefit
f ☐ Student
g ☐ Other

LIVING SITUATION

f167 I vilken typ av område bor du?

**1** ☐	Storstad: centralt	**5** ☐	Större tätort
**2** ☐	Storstad: ytterområde/förort	**6** ☐	Mindre tätort
**3** ☐	Stad: centralt	**7** ☐	Ren landsbygd
**4** ☐	Stad: ytterområde		

In English: What type of area do you live in?

1 ☐ Large city: central
2 ☐ Large city: outer area/suburb
3 ☐ Town/city: central
4 ☐ Town/city: outer area
5 ☐ Larger town
6 ☐ Smaller town
7 ☐ Rural area

FINALLY, A FEW QUESTIONS ABOUT YOURSELF

(When we compile the results from the SOM surveys, we usually present the answers in different groups. Therefore, we need to ask you a few final questions).

f170 Är du:

☐ Kvinna	☐ Man	☐ Annat: ...**ansex**...
**1**	**2**	**3**

f171 Vilket år är du född?

Årtal: [| | |]

f172 Är du:

Svensk medborgare	Medborgare i annat land	Både svensk medborgare och medborgare i annat land
☐	☐	☐
**1**	**2**	**3**

f173 Är du?

Ensamstående/singel	I ett förhållande	Sambo	Gift/i registrerat partnerskap	Änka/änkling
☐	☐	☐	☐	☐
**1**	**2**	**3**	**4**	**5**

In English:

f170 Are you:

☐ Woman ☐ Man ☐ Other: …………………….

---

f171 What year were you born?

Year: ☐☐☐☐

---

f172 Are you:

☐ A Swedish citizen ☐ A citizen of another country

☐ Both a Swedish citizen and a citizen of another country

---

f173 Are you:

☐ Single ☐ In a relationship ☐ Cohabiting ☐ Married / in a registered partnership

☐ Widowed

---

f176 Hur ser ditt hushåll ut?

				Ja	Nej
**a**	**1** ☐ Jag bor ensam ⟶ *Gå till fråga XX*				
	**2** ☐ Jag bor med/delar regelbundet mitt hushåll med: ⟶	**ba**	En vuxen	☐	☐
		**bb**	Flera vuxna	☐	☐
		**bc**	Ett eller flera barn	☐	☐
				**1**	**2**

In English: What is your household situation?

1 ☐ I live alone

2 ☐ I live with / regularly share my household with:

One adult ☐ Yes ☐ No

Several adults ☐ Yes ☐ No

One or more children ☐ Yes ☐ No

f179 Vilket språk talas huvudsakligen i ditt nuvarande hem?

☐ Svenska **1** ☐ Annat språk: ......**anspraak.**................................ **2**

In English: What language is mainly spoken in your current home?

☐ Swedish ☐ Another language: ……………………

f180 Vilken är din högsta skolutbildning?

1 ☐ Grundskola eller motsvarande, kortare än 9 år
2 ☐ Grundskola eller motsvarande, 9 år eller längre

3 ☐ Gymnasium eller motsvarande, kortare än 3 år
4 ☐ Gymnasium eller motsvarande, 3 år eller längre

5 ☐ Eftergymnasial utbildning, ej högskola/universitet, kortare än 3 år
6 ☐ Eftergymnasial utbildning, ej högskola/universitet, 3 år eller längre

7 ☐ Högskola/universitet, kortare än 3 år
8 ☐ Högskola/universitet, 3 år eller längre men kortare än 4 år
9 ☐ Högskola/universitet, 4 år eller längre

10 ☐ Forskarutbildning

In English: What is your highest level of education?

1 ☐ Primary school or equivalent, less than 9 years
2 ☐ Primary school or equivalent, 9 years or more
3 ☐ Upper secondary school or equivalent, less than 3 years
4 ☐ Upper secondary school or equivalent, 3 years or more
5 ☐ Post-secondary education (not university), less than 3 years
6 ☐ Post-secondary education (not university), 3 years or more
7 ☐ University/college, less than 3 years
8 ☐ University/college, 3 years or more but less than 4 years
9 ☐ University/college, 4 years or more
10 ☐ Doctoral education (PhD or equivalent)
