# Supplementary material for: Public understanding of palliative care and preferences for place of end-of-life care and death: A national population-based latent class analysis
Source: Palliat Care Soc Pract. 2026 Jul 29;20:26323524261474030. doi: 10.1177/26323524261474030 (PMC13424512; doi:10.1177/26323524261474030)
Supplement: Supplemental material - Public understanding of palliative care and preferences for place of end-of-life care and death: A national population-based latent class analysis [file sj-pdf-2-pcr-10.1177_26323524261474030.pdf]

Supplementary Table S1. Model fit indices for latent class analysis (n = 1,752).

No. of classes	BIC	AIC	Log-likelihood	Pearson χ² Goodness-of-fit	df.	Likelihood ratio/ deviance statistic	Relative entropy
1	28146	28015	−13984	245123	24	6903	NA
2	25709	25441	−12671	74326	49	4279	0.86
3	25398	24994	−12423	55967	74	3782	0.72
4	25232	24691	−12246	41421	99	3429	0.79
5	25175	24497	−12124	25986	124	3185	0.77
6	25248	24434	−12068	18306	149	3072	0.76

The five-class solution showed the lowest BIC and was selected as the final model for subsequent analyses.

Relative entropy quantifies classification certainty, with higher values indicating clearer class separation: 0.90 = very strong separation; 0.80–0.89 = good; 0.70–0.79 = moderate; and < 0.70 = weak separation. Entropy is not defined for a one-class model (NA).

Abbreviations: BIC, Schwarz's Bayesian Information Criterion; AIC, Akaike information criterion; df, degrees of freedom; NA, not applicable.

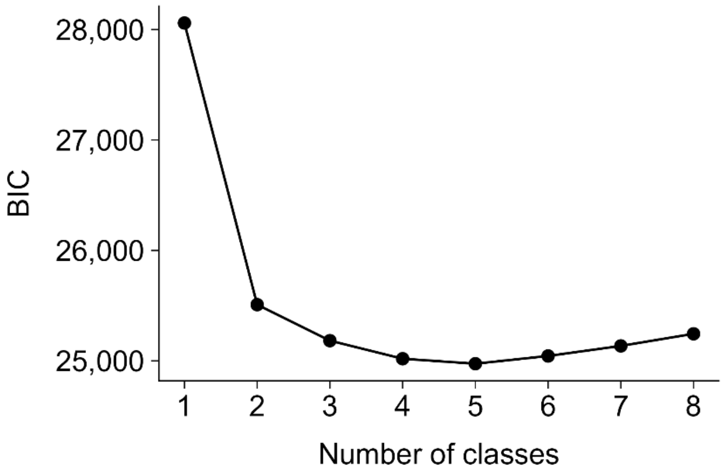

Supplementary Figure S1. Model fit assessed by using the Bayesian information criterion (BIC) for latent class solutions grouping participants by palliative care understanding, with models ranging from on one to eight latent classes.

Supplementary Table S2. Distribution of responses to six statements on palliative care understanding across five latent classes (n = 1,752).

	Comprehensive understanding (n=507)	Some understanding (n=584)	Limited understanding (n=283)	Misunderstanding (n=57)	No opinion (n=321)
Palliative care aims to reduce the patient's suffering					
Strongly disagree	2 (0.4%)	3 (0.5%)	2 (0.7%)	23 (40.4%)	0 (0.0%)
Disagree	1 (0.2%)	8 (1.4%)	16 (5.7%)	2 (3.5%)	0 (0.0%)
Agree	16 (3.2%)	133 (22.8%)	121 (42.8%)	6 (10.5%)	7 (2.2%)
Strongly agree	485 (95.7%)	411 (70.4%)	144 (50.9%)	25 (43.9%)	32 (10.0%)
No opinion	3 (0.6%)	29 (5.0%)	0 (0.0%)	1 (1.8%)	282 (87.9%)
Palliative care hastens death					
Strongly disagree	349 (68.8%)	161 (27.6%)	31 (11.0%)	36 (63.2%)	1 (0.3%)
Disagree	92 (18.1%)	123 (21.1%)	132 (46.6%)	6 (10.5%)	0 (0.0%)
Agree	33 (6.5%)	74 (12.7%)	105 (37.1%)	0 (0.0%)	0 (0.0%)
Strongly agree	22 (4.3%)	17 (2.9%)	13 (4.6%)	15 (26.3%)	1 (0.3%)
No opinion	11 (2.2%)	209 (35.8%)	2 (0.7%)	0 (0.0%)	319 (99.4%)
Palliative care is provided in all types of healthcare and care facilities					
Strongly disagree	96 (18.9%)	9 (1.5%)	27 (9.5%)	31 (54.4%)	0 (0.0%)
Disagree	69 (13.6%)	11 (1.9%)	93 (32.9%)	2 (3.5%)	0 (0.0%)
Agree	96 (18.9%)	38 (6.5%)	133 (47.0%)	3 (5.3%)	0 (0.0%)
Strongly agree	140 (27.6%)	12 (2.1%)	9 (3.2%)	5 (8.8%)	2 (0.6%)
No opinion	106 (20.9%)	514 (88.0%)	21 (7.4%)	16 (28.1%)	319 (99.4%)
Morphine at the end of life alleviates pain without hastening death					
Strongly disagree	292 (57.6%)	132 (22.6%)	44 (15.5%)	35 (61.4%)	19 (5.9%)
Disagree	93 (18.3%)	109 (18.7%)	102 (36.0%)	9 (15.8%)	15 (4.7%)
Agree	76 (15.0%)	96 (16.4%)	108 (38.2%)	5 (8.8%)	16 (5.0%)
Strongly agree	31 (6.1%)	37 (6.3%)	17 (6.0%)	7 (12.3%)	5 (1.6%)
No opinion	15 (3.0%)	210 (36.0%)	12 (4.2%)	1 (1.8%)	266 (82.9%)
Pain is inevitable in the dying process					
Strongly disagree	20 (3.9%)	24 (4.1%)	6 (2.1%)	39 (68.4%)	7 (2.2%)
Disagree	23 (4.5%)	13 (2.2%)	54 (19.1%)	3 (5.3%)	0 (0.0%)
Agree	113 (22.3%)	135 (23.1%)	161 (56.9%)	4 (7.0%)	20 (6.2%)

Strongly agree	333 (65.7%)	167 (28.6%)	55 (19.4%)	9 (15.8%)	31 (9.7%)
No opinion	18 (3.6%)	245 (42.0%)	7 (2.5%)	2 (3.5%)	263 (81.9%)
Palliative care includes support for family members					
Strongly disagree	3 (0.6%)	8 (1.4%)	7 (2.5%)	46 (80.7%)	5 (1.6%)
Disagree	1 (0.2%)	15 (2.6%)	53 (18.7%)	2 (3.5%)	1 (0.3%)
Agree	75 (14.8%)	122 (20.9%)	150 (53.0%)	1 (1.8%)	9 (2.8%)
Strongly agree	404 (79.7%)	158 (27.1%)	56 (19.8%)	2 (3.5%)	9 (2.8%)
No opinion	24 (4.7%)	281 (48.1%)	17 (6.0%)	6 (10.5%)	297 (92.5%)

Descriptive statistics are presented as counts and column percentages within each latent class. Percentages may not total 100% due to rounding.

Supplementary Table S3. Background characteristics of five latent classes identified according to perceptions of palliative care using latent class analysis (n=1,752).

	Comprehensive understanding (n=507)	Some understanding (n=584)	Limited understanding (n=283)	Misunderstanding (n=57)	No opinion (n=321)
Gender					
Women	306 (60.4%)	322 (55.1%)	118 (41.7%)	23 (40.4%)	149 (46.4%)
Men	192 (37.9%)	255 (43.7%)	160 (56.5%)	33 (57.9%)	169 (52.6%)
Other	9 (1.8%)	7 (1.8%)	5 (1.8%)	1 (1.8%)	3 (0.9%)
Age (years), mean (SD)	57 (17)	55 (20)	55 (18)	58 (21)	50 (22)
Citizenship					
Swedish	461 (90.9%)	542 (92.8%)	258 (91.2%)	50 (87.7%)	296 (92.2%)
Dual citizenship (incl. Swe)	27 (5.3%)	27 (4.6%)	12 (4.2%)	1 (1.8%)	15 (4.7%)
Other citizenship	19 (3.7%)	15 (2.6%)	13 (4.6%)	6 (10.5%)	10 (3.1%)
In a relationship	400 (78.9%)	433 (74.1%)	216 (76.3%)	34 (59.6%)	213 (66.4%)
Living alone	93 (18.3%)	111 (19.0%)	59 (20.8%)	16 (28.1%)	77 (24.0%)
Swedish as primary language	479 (94.5%)	551 (94.3%)	258 (91.2%)	47 (82.5%)	296 (92.2%)
Education level					
Primary (9 years)	58 (11.4%)	83 (14.2%)	36 (12.7%)	11 (19.3%)	79 (24.6%)
Secondary (12 years)	192 (37.9%)	258 (44.2%)	120 (42.4%)	26 (45.6%)	139 (43.3%)
University (>12 years)	257 (50.7%)	243 (41.6%)	127 (44.9%)	20 (35.1%)	103 (32.1%)
Residential area					
Small city, or rural area	53 (10.5%)	62 (10.6%)	41 (14.5%)	11 (19.3%)	41 (12.8%)
City	283 (55.8%)	296 (50.7%)	160 (56.5%)	29 (50.9%)	170 (53.0%)
Big city	171 (33.7%)	226 (38.7%)	82 (29.0%)	17 (29.8%)	110 (34.3%)
Work status					
Employed	284 (56.0%)	288 (49.3%)	157 (55.5%)	23 (40.4%)	146 (45.5%)
Unemployed	4 (0.8%)	15 (2.6%)	7 (2.5%)	2 (3.5%)	10 (3.1%)
Retired	182 (35.9%)	226 (38.7%)	98 (34.6%)	24 (42.1%)	100 (31.2%)
Long-term sick leave	11 (2.2%)	12 (2.1%)	4 (1.4%)	1 (1.8%)	12 (3.7%)
Student	30 (5.9%)	56 (9.6%)	20 (7.1%)	4 (7.0%)	56 (17.4%)
Other	15 (3.0%)	9 (1.5%)	14 (4.9%)	6 (10.5%)	7 (2.2%)
Overall health status*, median (IQR)	2 (1–2)	2 (1–2)	2 (1–2)	2 (1–2)	2 (1–2)
Overall life satisfaction†, median (IQR)	8 (6–9)	8 (6–8)	7 (6–8)	8 (6–9)	8 (6–9)

Descriptive data are presented as means with standard deviation (SDs) for continuous variables, medians with interquartile ranges (IQRs) for ordinal variables, and counts and percentages for other categorical variables.
* Overall health status was rated on a scale from 0 (very poor) to 10 (very good).
† Overall life satisfaction was rated on a scale from 1 (very satisfied) to 4 (very dissatisfied).
Abbreviations: IQR, interquartile range; SD, standard deviation; Swe, Swedish.

Supplementary Table S4. Participant characteristics associated with latent class membership in multivariable multinomial logistic regression (n = 1,752). Results are presented as odds ratios (ORs) with 95% confidence intervals (CIs) for belonging to Classes 2–5 versus Class 1.

	Class 2 vs. 1	Class 3 vs. 1	Class 4 vs. 1	Class 5 vs. 1
Women vs. Men	0.85 (0.62, 1.16)	0.58 (0.40, 0.84)	0.55 (0.28, 1.10)	0.58 (0.41, 0.83)
Age (per 20 years increase)	0.78 (0.58, 1.04)	0.82 (0.58, 1.15)	1.08 (0.56, 2.06)	0.59 (0.42, 0.83)
Citizenship				
Dual citizenship (incl. Swedish) vs. Swedish	0.74 (0.34, 1.63)	0.51 (0.18, 1.45)	0.09 (0.00, 121.5)	0.70 (0.28, 1.76)
Non-Swedish vs. Swedish	0.88 (0.28, 2.80)	1.02 (0.31, 3.39)	1.58 (0.26, 9.55)	0.76 (0.22, 2.61)
In a relationship	0.77 (0.45, 1.32)	1.24 (0.64, 2.40)	0.37 (0.12, 1.14)	0.82 (0.46, 1.47)
Heterosexual	1.15 (0.54, 2.46)	0.74 (0.33, 1.66)	1.38 (0.29, 6.68)	0.75 (0.36, 1.56)
Living alone	0.94 (0.53, 1.68)	1.40 (0.71, 2.76)	0.66 (0.20, 2.17)	1.32 (0.72, 2.44)
Swedish as primary language	0.72 (0.30, 1.70)	0.51 (0.20, 1.27)	0.28 (0.06, 1.27)	0.56 (0.23, 1.37)
Level of education				
Primary vs. University	1.29 (0.74, 2.26)	1.34 (0.69, 2.59)	2.29 (0.82, 6.43)	3.78 (2.10, 6.79)
Secondary vs. University	1.48 (1.05, 2.07)	1.37 (0.92, 2.06)	1.32 (0.60, 2.92)	1.88 (1.25, 2.81)
Geographic area				
Urban vs. City	1.29 (0.76, 2.19)	1.22 (0.67, 2.22)	1.22 (0.40, 3.79)	1.20 (0.66, 2.19)
Big city vs. City	1.21 (0.87, 1.69)	0.80 (0.53, 1.21)	0.82 (0.39, 1.74)	1.00 (0.68, 1.47)
Occupation				
Unemployed vs. Employed	1.85 (0.36, 9.39)	1.15 (0.17, 7.57)	1.67 (0.02, 125.4)	1.70 (0.33, 8.84)
Retiree vs. Employed)	1.60 (0.94, 2.72)	1.21 (0.64, 2.29)	1.14 (0.35, 3.67)	1.28 (0.68, 2.42)
Long-term sick leave vs. Employed	0.79 (0.27, 2.33)	0.08 (0.00, 36.2)	0.80 (0.09, 7.12)	1.25 (0.43, 3.58)
Student vs. Employed	1.26 (0.61, 2.62)	0.88 (0.35, 2.21)	0.49 (0.05, 5.30)	1.77 (0.85, 3.71)
Other vs. Employed	0.73 (0.21, 2.48)	2.65 (0.88, 7.98)	5.72 (1.39, 23.6)	0.69 (0.16, 2.99)
Overall satisfaction in life *	0.99 (0.75, 1.31)	1.27 (0.91, 1.77)	1.50 (0.81, 2.79)	1.03 (0.74, 1.42)
Overall health status †	0.98 (0.90, 1.07)	0.96 (0.87, 1.07)	1.04 (0.86, 1.27)	1.01 (0.91, 1.12)

Statistical analyses were conducted using multivariable multinomial logistic regression estimated with a bias-corrected three-step procedure accounting for classification uncertainty. Class 1 (Comprehensive understanding) served as the reference category. ORs >1 indicate a higher and ORs <1 a lower likelihood of membership compared with Class 1.
Class 1 = Comprehensive understanding; Class 2 = Some understanding; Class 3 = Limited understanding; Class 4 = Misunderstanding; Class 5 = No opinion.
* Life satisfaction was measured on a 1–4 scale, with lower values indicating higher satisfaction (1 = very satisfied, 4 = very dissatisfied).
† Self-rated health was measured on a 0–10 scale, with higher values indicating better perceived health (0 = very poor, 10 = very good).

Supplementary Table S5. Preferred place of care and place of death across latent classes (n = 1,752).

	Comprehensive understanding (n=507)	Some understanding (n=584)	Limited understanding (n=283)	Misunderstanding (n=57)	No opinion (n=321)
**Preferred place of care at the end of life**					
At home	56.7%	60.4%	59.5%	50.4%	61.8%
At a friend's home	8.9%	10.6%	10.4%	6.8%	15.3%
In a hospice or palliative care unit	16.3%	10.4%	10.1%	13.4%	6.4%
In hospital	5.8%	6.1%	7.6%	11.0%	5.8%
In a care facility	8.8%	8.3%	6.9%	11.9%	6.4%
Somewhere else	3.5%	4.3%	5.5%	6.5%	4.3%
**Preferred place of death**					
At home	50.1%	55.8%	55.0%	42.0%	58.0%
At a friend's home	8.4%	10.4%	8.5%	10.6%	15.0%
In a hospice or palliative care unit	22.2%	12.5%	12.7%	18.2%	5.6%
In hospital	7.3%	7.5%	7.6%	12.4%	8.9%
In a care facility	7.7%	8.5%	8.1%	12.4%	5.5%
Somewhere else	4.4%	5.3%	8.1%	4.2%	7.1%

Respondents were asked: *"Imagine you had a serious illness and less than a year to live, where would you prefer to receive care?"* and *"Imagine you had a serious illness and less than a year to live, where would you prefer to die?"* Responses were rated on a four-point Likert scale ("strongly disagree" to "strongly agree") with an additional "no opinion" option.
The preferred place of care and death was defined as the location(s) with the highest level of agreement on the Likert scale, assigning equal weights when ties occurred. Percentages represent the share of total weighted votes within each latent class.
